# Editorial: Exploring KLF4’s role in immune cell function and disease progression

**DOI:** 10.3389/fimmu.2025.1672030

**Published:** 2025-08-18

**Authors:** H. Daniel Lacorazza, Walden Ai

**Affiliations:** ^1^ Baylor College of Medicine, Texas Children’s Hospital, Houston, TX, United States; ^2^ Department of Biology, Benedict College, Columbia, SC, United States

**Keywords:** KLF4, trained immunity, immune cells, alternative splicing, inflammation, stem cells

Early work on the transcription factor Krüppel-like factor 4 (KLF4) was focused on its function in epithelial and endothelial cells, which has been extensively studied. The groundbreaking discovery of induced pluripotent stem cells reignited interest in KLF4, positioning it as a central regulator of stem cell biology. Moreover, emerging evidence highlights its important role in immune cells, especially in inflammation, tissue homeostasis, and disease development. For example, KLF4 is upregulated during the generation of exhausted memory monocytes (1). Consistently, KLF4 mediates efferocytosis, which is linked to enhanced injury resolution through trained immunity in alveolar macrophages (2). This Research Topic aims to elucidate the specific roles of KLF4 within the immune system, including its involvement in trained immunity, inflammation, cancer development, and potential molecular mechanisms.

The recent review by Das et al. provides a comprehensive overview of the multifaceted role of KLF4 in immune cell regulation. Within the innate immune system, KLF4 promotes monocytic differentiation, modulates macrophage polarization toward both M1 and M2 phenotypes, facilitates granule formation and TLR4 downregulation in neutrophils, and supports dendritic cell differentiation as well as natural killer (NK) cell survival. Consequently, loss of KLF4 impairs host defense mechanisms, particularly against bacterial pathogens. In the adaptive immune system, KLF4 negatively regulates the proliferation of CD8^+^ T cells and B cells and inhibits the differentiation of Th17 CD4^+^ T cells. Through these diverse regulatory functions, KLF4 significantly influences immune responses across a broad spectrum of infectious and inflammatory disease models.

Lacorazza provides an in-depth analysis of the context-dependent role of KLF4 in hematopoietic stem cells (HSCs), leukemia, and immune regulation. In murine leukemia models, KLF4 exhibits dual functionality, acting as both a tumor suppressor and a pro-oncogene, depending on the leukemic context. Specifically, KLF4 inhibits the expansion of leukemia stem/initiating cells in T-cell acute lymphoblastic leukemia (T-ALL), while preserving the regenerative potential of normal HSCs by repressing toll-like receptor signaling and the non-canonical NFκB2 pathway. In contrast, KLF4 supports the self-renewal capacity of leukemic stem/initiating cells in chronic myeloid leukemia (CML) by downregulating a key inhibitory pathway, as well as in acute myeloid leukemia (AML). These findings position KLF4 as a critical molecular switch in hematologic malignancies, underscoring its potential as a therapeutic target for selective intervention.

Ju et al. examine the multifaceted role of KLF4 in both solid tumors and hematological malignancies, highlighting its influence through intrinsic tumor mechanisms as well as modulation of the tumor microenvironment and immune surveillance. KLF4 exhibits dual functionality in solid tumors, acting as a tumor suppressor in hepatocellular carcinoma and gastric cancer, while promoting oncogenic activity in low-grade primary ductal carcinoma, prostate cancer, colorectal cancer, and lung cancer. Notably, beyond its cell-intrinsic roles, the review highlights the impact of KLF4 on immune cell populations, including T cells and macrophages, which shape the tumor immune microenvironment by suppressing immune responses and modulating inflammatory signaling. These insights underscore the significance of KLF4 as a key regulator of tumor biology and immunotherapy.

Herta et al. reviewed a key role of KLF4 in phagocytes, including neutrophils, monocytes, macrophages, and dendritic cells, bridging innate and adaptive immunity. KLF4 functions as a regulator of phagocyte differentiation, polarization, and inflammatory modulation. As noted by the earlier reviewers, its role is highly context-dependent, exhibiting both pro-and anti-inflammatory properties based on environmental signals, cellular states, and the invading pathogen. Its diverse involvement in immune responses suggests that it contributes to maintaining a balance between effective pathogen defense and the prevention of excessive and potentially harmful inflammation. This review also discussed potential implications for therapeutic interventions targeting KLF4.

At a molecular level, Rosewell Shaw et al. proposed that different KLF4 isoforms through alternative splicing may be responsible for its diverse and even contradictory roles in immune regulation such as M1 and M2 macrophage polarization. Additionally, although the heterogeneity of breast cancer stem cells has been reported, whether or not different KLF4 isoforms are involved in the dynamics of breast cancer stem cells remains elusive. Moreover, the phenomenon of trained immunity is a form of innate immune memory marked by epigenetic and metabolic reprogramming. This review presents current knowledge of KLF4 alternative splicing in myeloid cells and explores novel connections for how KLF4 isoform diversity may contribute to cellular plasticity and differential immune responses of myeloid cells across physiological and pathological conditions.

KLF4 does not function in isolation. Rather, it synergizes with other members of the Kruppel-like factor (KLF) family. In hematopoiesis, KLFs have essential roles in myeloid cell differentiation and function. Salmon et al. reviewed that KLF4, together with KLF2, KLF3 and KLF6, regulates macrophage development and initiates pro-and anti-inflammatory signaling pathways in response to various stimuli. They reviewed how KLFs cooperate and compete to either activate or repress target genes to influence initiation and resolution of inflammatory responses in macrophages. They further discussed how KLFs may be involved in the development of chronic inflammatory conditions.

The objective of the Research Topic is to address key questions regarding KLF4’s involvement and to explore the relevant hypotheses in both innate and adaptive immune cells. By exploring these aspects from the Research Topic, we hope future research will deepen our understanding of KLF4’s contribution to immunology-related processes and its potential implications for therapeutic interventions.
